# miR-494 protects pancreatic β-cell function by targeting PTEN in gestational diabetes mellitus

**DOI:** 10.17179/excli2017-491

**Published:** 2017-12-12

**Authors:** Yanfang He, Jie Bai, Ping Liu, Jianxin Dong, Yajuan Tang, Jianli Zhou, Ping Han, Jun Xing, Yan Chen, Xiangyang Yu

**Affiliations:** 1 Department of Obstetrics and Gynecology; Affiliated Hospital of North China University of Science and Technology, Tangshan 063000, China

**Keywords:** gestational diabetes mellitus, microRNA-494, pancreatic beta-cells, PTEN

## Abstract

Gestational diabetes mellitus (GDM) is one of the most common pregnancy complications characterized by insulin resistance and pancreatic β-cell dysfunction. Increasing evidence suggests that microRNAs (miRNAs) play key roles in the diverse types of diabetes, including GDM. However, the underlying mechanisms remain largely unknown. The purpose of this study is to investigate the role of microRNAs in GDM. The microarray data of dysregulated miRNAs in blood and placenta was retrieved in the GEO dataset under the accession number GSE19649. Quantitative reverse transcription PCR (qRT-PCR) was performed to analyze the expression levels of miR-494 in peripheral blood from twenty pairs of gestational diabetes (GDM) women and healthy women. Then, we investigated the effects of miR-494 on the insulin secretion of pancreatic β-cells. Moreover, the role of this miR-494 in regulating the proliferation and apoptosis of pancreatic β-cells were determined by MTT assay and flow cytometry, respectively in INS1 cells transfected with a miR-494 mimic or inhibitor. In addition, the direct target of miR-494 was confirmed using 3' untranslated region (UTR) luciferase reporter assay. Our data demonstrated that the miR-494 level was significantly decreased in the blood of GDM patients, and the low level was associated with a high concentration of blood glucose. Furthermore, overexpression of miR-494 improved pancreatic β-cell dysfunction by enhancing insulin secretion and total insulin content, inducing cell proliferation, and inhibiting cell apoptosis, whereas miR-494 knockdown exhibited decreased insulin secretion and proliferation, as well as stimulated apoptosis. In addition, phosphatase and tensin homolog (PTEN) which has been shown to play a pivotal role in apoptosis, was proved to be a direct target of miR-494 in pancreatic β-cells. More importantly, siRNA-induced downregulation of PTEN reversed the effects of miR-494 knockdown on insulin secretion, cell proliferation, and apoptosis of pancreatic β-cells.

## Introduction

 Gestational diabetes mellitus (GDM) is one of the most common complications in pregnancy and affects 3-8 % of all pregnancies (Barnes-Powell, 2007[4], Boucher et al., 2011[8]). Both women diagnosed with GDM and their offspring are at enhanced risk for developing diabetes mellitus. Previous studies have shown that GDM is associated with pancreatic β-cell dysfunction (Barbour et al., 2007[3]). However, the specific mechanism through which β-cell dysfunction occurs is extremely complicated and remains unclear.

Pancreatic β-cell dysfunction is a central component of the pathogenesis of GDM (Chen et al., 2015[10]). Decline in β-cell function is characterized by a reduction of β-cell mass caused by an increased apoptosis rate and defective insulin production and secretion from the remaining β-cells (Weir et al., 2001[37]; Butler et al., 2003[9]; Maedler and Donath, 2004[23]; Prentki and Nolan, 2006[27]). It has been suggested that systemic insulin-resistance, low grade inflammation, chronic excess of cholesterol and of metabolic fuels could trigger β-cell damage over time, especially in genetically predisposed individuals (Vrachnis et al., 2012[35]). In GDM, pancreatic β-cell dysfunction as well as insulin resistance are altered, and the pathophysiologic mechanisms underlying these changes are not completely understood. 

MicroRNAs (miRNAs), a class of non-coding RNAs, 18-25 nucleotides in length, could negatively regulate gene expression at post-transcriptional level by directly binding to the 3´-untranslated region (3´-UTR) of target mRNA to induce mRNA deregulation or translational repression (Ambros, 2004[1]). As a large family of gene regulators, miRNAs are closely associated with various cellular processes, such as proliferation, apoptosis, differentiation and metabolism (Bartel, 2004[5]; Bhatia et al., 2015[7]). Many miRNAs have been demonstrated to participate in the etiology and pathogenesis of diabetes mellitus and its complications (Latreille et al., 2015[17]; Martinez-Sanchez et al., 2015[24]). For example, Jacovetti et al. (2015[16]) found that β-cell maturation was associated with alterations in the expression of miRs induced by the nutritional transition. A recent study reported that miR-101a and miR-30b contribute to inflammatory cytokine-mediated β-cell dysfunction (Zheng et al., 2015[41]). However, limited studies have paid attention on miRNAs and their roles in GDM.

In this study, we found that miR-494 was significantly down-regulated in GDM patients, and played an important role in the regulation of pancreatic β-cell proliferation, apoptosis and insulin secretion through targeting PTEN. Accordingly, we suggest that miR-494 may serve as a potential candidate for the clinical management of GDM.

## Materials and Methods

### MicroRNA expression profile data from Gene Expression Omnibus (GEO)

MicroRNA array expression profile data GSE19649 were downloaded from open Gene Expression Omnibus (GEO) database (https://www.ncbi.nlm.nih.gov/geo/). These microRNA array expression data were analyzed by GEO2R bioinformatics software (http://www.ncbi.nlm.nih.gov/geo/geo2r/), which is an interactive web tool for comparing two groups of data that can analyze any GEO series. The data were analyzed using limma (Smyth, 2004[30]) and significance analysis of microarray (SAM) (Tusher et al., 2001[34]), to determine differential expression of microRNAs between groups. MicroRNAs were identified as significant based upon the P < 0.05 with limma analysis and a median FDR < 0.05 with SAM. Heat map of differentially expressed genes (DEGs) was generated using the online tool Morpheus (https://software.broadinstitute.org/morpheus/).

### Blood sample collection 

This study was approved by the Ethics Committee of the Affiliated Hospital of North China University of Science and Technology, and was carried out in accordance with the Declaration of Helsinki. Written informed consent was obtained from all study subjects prior to enrollment. Blood samples from patients with GDM (n=20) and normal subjects (n=20) were collected from the Affiliated Hospital of North China University of Science and Technology during November, 2014 to February, 2015. Patients with serious liver or kidney diseases, malignancy and acute heart failure were excluded.

### Cell culture and transfection 

The INS-1 cell line, a well differentiated mouse insulinoma β-cell line was maintained in Dulbecco's modifed Eagle's medium containing high glucose (Sigma-Aldrich, St. Louis, MO, USA), supplemented with 10 % heat-inactivated fetal bovine serum (Gibco) and 70 µM β-mercaptoethanol, at 37 °C in a humidified atmosphere of 5 % CO_2_.

MiR-494 mimics, miR-494 inhibitor and controls were purchased from Shanghai GenePharma (Shanghai, China). INS-1 cells were transfected with 20 nM miR-494 mimic, miR-494 inhibitor or NC miRNAs with Lipofectamine™ RNAiMAX (Life Technologies, Grand Island, NY, USA). 48 hours after transfection, cells were collected for further experiments.

### RNA isolation and qRT-PCR

Total RNA isolation from peripheral blood was performed using Trizol reagent (Invitrogen, Carlsbad, CA, USA) according to the manufacturer's instructions. The expression level of mature miR-494 in blood samples was detected by qRT-PCR and calculated as described (Feng et al., 2010[13]). U6 was used for the normalization of miR-494 expression. The expression level of PTEN mRNA was measured by qRT-PCR according to the Taqman1 Gene Expression Assays (Applied Biosystems, Foster City, CA, USA). The relative expression of PTEN mRNA compared with GAPDH mRNA was calculated using the 2^-Δ^CT method. The following primers were used: PTEN mRNA forward: 5´-AAGCTGGAAAGGGACGAACT-3´, reverse: 5´-ACACATAGCGCCTCTGACTG-3´ and GAPDH mRNA forward: 5´-CCTCTCTCTAATCAGCCCTCTG-3´, reverse: 5´-AGAAGGCTGGGGCTCATTTG-3´ (Sangon, Shanghai, China).

### Detection of glucose-stimulated insulin secretion

The cells were seeded in a 96-well plate and cultured for 24 h. The cells were then treated with basal glucose (3.3 mM) or stimulatory glucose (16.7 mM) for 1 h. Subsequently, the insulin level was measured by ELISA. Total insulin content was measured after sonication of cells in acid ethanol (2 % H_2_SO_4_), followed by 3 freeze/thaw cycles, and then centrifuged for 5 min at 10,000 × g. The supernatant was used to measure the insulin level by ELISA as described above.

### Cell viability assay

Cultured or transfected cells were seeded in a 96-well plate (2×10^3^ cells/well) in 100 μL growth medium. After 24 h incubation at 37 °C, 20 μl of MTT solution (5 mg/ml) was added to each well, and the cells were continuously incubated for 4 h. After incubation, 200 μl of DMSO was added to each well to dissolve the formazan formed and the absorbance was read with a microplate reader (BioTek, Richmond, USA) at 490 nm according to the manufacturer's instructions.

### Apoptosis assay

Cultured or transfected cells were collected, washed with PBS and resuspended in binding buffer containing Annexin V-FITC and propidium iodide (Pitha-Rowe et al., 2009[26]). After 15 min of incubation at room temperature, samples were analyzed on a FACSalibur flow cytometer to determine rate of apoptosis.

### Luciferase reporter assay

A whole fragment of 3´UTR PTEN-5 mRNA and a mutant form were cloned into pGL-3-Luc. The INS-1 cells were seeded in 12-well plates and co-transfected with pGL-3-PTEN-5 wild-type or mutant portion and TK100 Renilla combined with miR-494 mimic or NC control using Lipofectamine 2000 (Invitrogen). After 48 h of incubation, cells were collected for application in the Dual-Luciferase Reporter System (Promega, Madison, WI) following the manufacturer's recommendations. All of the dual-luciferase reporter assays were done in triplicate within each experiment, and three independent experiments were conducted.

### Western blot

Total cellular proteins were extracted using RIPA lysis buffer containing proteinase inhibitor (Sigma, USA). Concentrations of total cellular protein were determined using a BCA assay kit (Pierce, Rockford, IL, USA). Total protein samples (40 μg) were analyzed by 8 % SDS-PAGE gel and transferred to polyvinylidene difluoride (PVDF) membranes by a wet blotting procedure (100 V, 120 mins, 4 °C). After blocking with 5 % blocking buffer, the membranes were incubated with primary antibodies against PTEN (1:500, Abcam, Cambridge, UK) at 4 °C overnight. Control antibodies were anti-β-actin (1:2000; Santa Cruz, CA, USA). The protein bands were visualized by enhanced chemiluminescence detection reagents (da Silva Xavier et al., 2003[11]) (Applygen Technologies Inc., Beijing, China) as described in the manufacturer's instructions. Relative band intensities were determined by densitometry using Scion image software (version 4.0).

### Statistical analysis

Statistical analyses were performed with SPSS 13.0 software. The results were evaluated by χ^2^ test and the other data were evaluated by Student's t-test and expressed as the mean ± SD from three independent experiments. A P-value of less than 0.05 was considered statistically significant.

## Results

### MiR-494 is significantly down-regulated in peripheral blood from GDM patients

**Figure 1:**
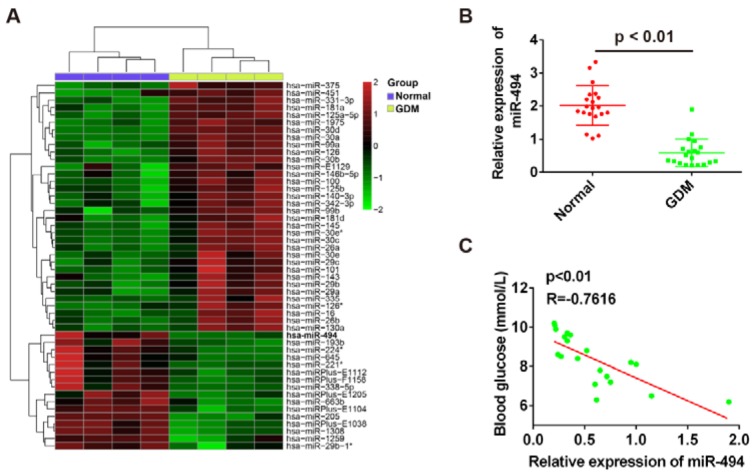
MiR-494 is significantly down-regulated in blood of GDM patients. (A) Differentially expressed miRNAs were analyzed using significance analysis of microarrays method. Data was retrieved from GEO dataset, with the accession number GSE19649. Expression values are represented in shades of red and green indicating expression above and below the median expression value across all samples. (B) The expression of miR-494 was measured by qRT-PCR in blood from 20 paired GDM women and healthy women, P < 0.01 vs the normal group. (C) Correlation between miR-494 level and blood glucose determined by Spearman correlation analysis (R = -0.7616, P < 0.01)

In this study, we first studied the dysregulated miRNAs in blood samples from GDM patients via retrieving the microarray data in the GEO dataset under the accession number GSE19649. The miRNA microarray identified 34 miRNAs that were up-regulated and 16 miRNAs that were down-regulated in blood samples between the GDM and Normal group. Among the aberrantly expressed miRNAs, we observed that miR-494 was one of the most being down-regulated miRNAs in GDM samples (Figure 1A(Fig. 1)). Since the pancreatic β-cell apoptosis contributes to impaired insulin secretion in GDM (Zhang et al., 2010[39]; Demirci et al., 2012[12]) and many previous studies showed that down-regulated expression of miR-494-3p can promote apoptosis in kinds of cells (Li et al., 2015[18]; Zhu et al., 2017[42]), we chose miR-494 for further study. To further verify the dysregulation, we further performed qRT-PCR analysis based on 20 paired GDM women and healthy women. The results showed that miR-494 was significantly downregulated in peripheral blood from GDM patients, when compared with that of normal subjects (Figure 1B(Fig. 1)). In addition, we further identified a negative correlation between the blood glucose concentration and miR-494 level among these GDM patients (R=-0.7616, p < 0.01) by performing linear regression analysis (Figure 1C(Fig. 1)). These data suggest that the down-regulation of miR-494 expression may be involved in the progression of GDM. 

### Knockdown of miR-494 inhibits insulin secretion and total insulin content

**Figure 2:**
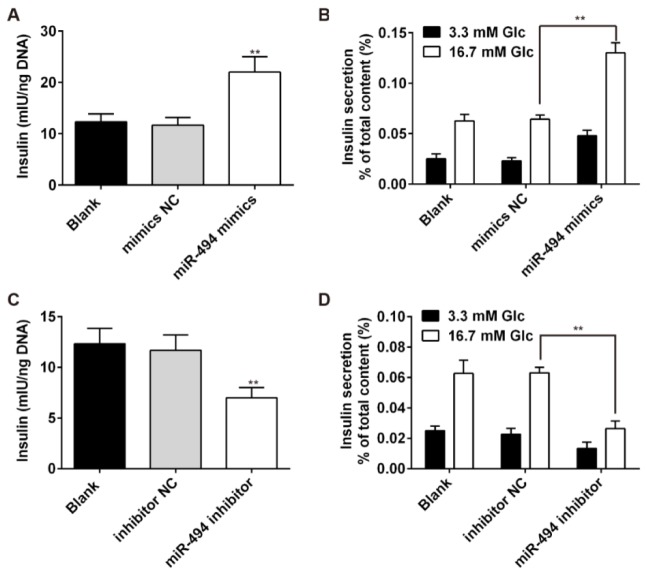
Knockdown of miR-494 inhibits the insulin secretion of pancreatic β-cells. (A and B) INS-1 cells were transfected with miR mimic (miR-NC) or miR-494 mimic. Glucose-stimulated (3.3 or 16.7 mmol glucose) insulin content and insulin secretion was then determined by ELISA assays. (C and D) INS-1 cells were transfected with the indicated miRNA inhibitor or a control inhibitor. Glucose-stimulated (3.3 or 16.7 mmol glucose) insulin content and insulin secretion was then determined by ELISA assays. All data are expressed as the mean ± SD. P < 0.01 vs mimic NC or inhibitor NC

The INS-1 cell line, a well differentiated mouse insulinoma β-cell line (Hohmeier et al., 2000[15]; Ronnebaum et al., 2008[29]) that displays the characteristics of pancreatic β-cells (including insulin secretion) in response to glucose stimulation was used in our *in vitro* experiments to investigate the function of miR-494 in regulating metabolism. As shown in Figure 2(Fig. 2), insulin secretion in response to glucose stimulus was increased in cells transfected with miR-494 mimic (Figure 2A, B(Fig. 2)), and decreased in cells transfected with miR-494 inhibitor (Figure 2C, D(Fig. 2)), when compared with the control and NC groups. These data indicated that miR-494 inhibited insulin secretion in pancreatic β-cells under conditions of glucose stimulation.

### Knockdown of miR-494 inhibits the proliferation and induces the apoptosis of pancreatic β-cells

**Figure 3:**
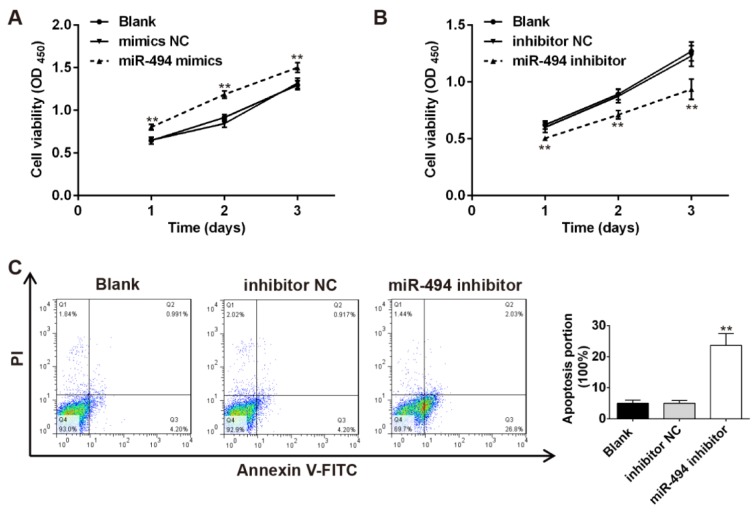
Knockdown of miR-494 inhibits the proliferation and induces the apoptosis of pancreatic β-cells. (A and B) Cell viability was measured by MTT assay after INS-1 cells transfected with miR-494 mimic or miR-494 inhibitor. (C) Cell apoptosis was performed by FCM after INS-1 cells transfected with miR-494 inhibitor. All data are expressed as the mean ± SD. *P < 0.01 vs mimic NC or inhibitor NC

Because the amount of secreted insulin is determined by the total number of β-cells in the pancreas (Chen et al., 2015[10]), we further investigate the effects of miR-494 on the proliferation and apoptosis of pancreatic β-cells by MTT assays and flow cytometry. The results showed that overexpression of miR-494 significantly promoted cell proliferation, when compared with the Blank and mimics NC groups (Figure 3A(Fig. 3)). Conversely, knockdown of miR-494 inhibited the proliferation and induces the apoptosis of pancreatic β-cells (Figure 3B, C(Fig. 3)). These data suggest that down-regulated miR-494 inhibits insulin secretion through suppressing the growth and promoting apoptosis of pancreatic β-cells.

### PTEN is a direct target of miR-494 in pancreatic β-cells

**Figure 4:**
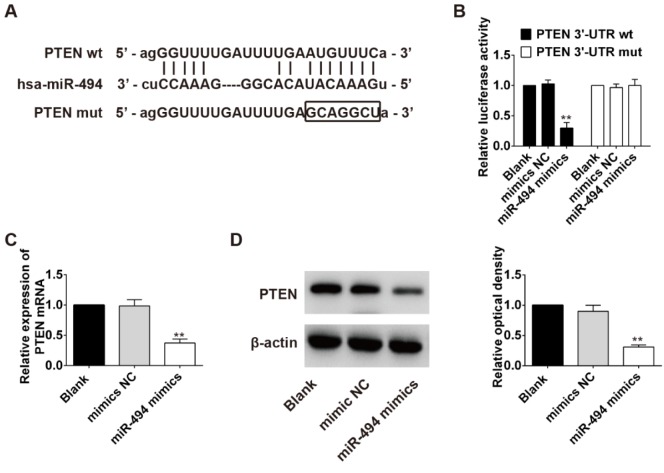
PTEN is a direct target of miR-494 in INS-1 cells. (A) Schema of the firefly luciferase reporter constructs for the PTEN, indicating the interaction sites between miR-494 and the 3´-UTRs of PTEN. (B) Luciferase activities. INS-1 cells were co-transfected with firefly luciferase constructs containing the PTEN wild-type or mutated 3´-UTRs and miR-494 mimic or mimics NC, as indicated (n=3). (C and D) mRMA levels and protein expression of PTEN after treatment with miR-494 mimic or mimics NC and the optical densities of the bands were measured using Image-Pro Plus software (n=3). All data are expressed as the mean ± SD. **P < 0.01 vs mimics NC

Several reports have highlighted the importance of PI3K/AKT signaling in pancreatic β-cell physiology (MacFarlane et al., 1994[22]; Rafiq et al., 2000[28]; Srinivasan et al., 2002[31]). It is well known that PI3K/AKT pathway can be regulated by PTEN through its lipid phosphatase and protein tyrosine phosphatase activity (Besson et al., 1999[6]; Pitha-Rowe et al., 2009[26]). Moreover, PTEN was also found to be a target of miR-494 in previous research (Sun et al., 2014[33]; Liu et al., 2015[21]; Yang et al., 2015[38]). Thus, we hypothesized that miR-494 exert its function in pancreatic β-cell by targeting PTEN, leading to regulation of PI3K/AKT pathway. Then, we used TargetScan software to analyze the putative target genes of miR-494. As expected, PTEN was predicated to be a potential target gene of miR-494 with evolutionary conservation (Figure 4A(Fig. 4)). 

To experimentally validate whether PTEN was a direct target of miR-494, we inserted the PTEN 3´-UTR transcript or a mutated version into a luciferase system. Transfection of the miR-494 mimic clearly decreased the luciferase activities of the 3´-UTR segment of PTEN, but the construct containing a mutant binding site abolished the inhibitory effect of the miR-494 mimic (Figure 4B(Fig. 4)). In addition, qRT-PCR analysis and Western blot analysis showed that miR-494 mimic decreased the levels of PTEN mRNA and protein expression in INS-1 cells (Figure 4C and D(Fig. 4)). These results indicated that PTEN was a direct target of miR-494 in INS-1 cells.

### PTEN is involved in the inhibitory effects of miR-494 knockdown on functions of pancreatic β-cell

**Figure 5:**
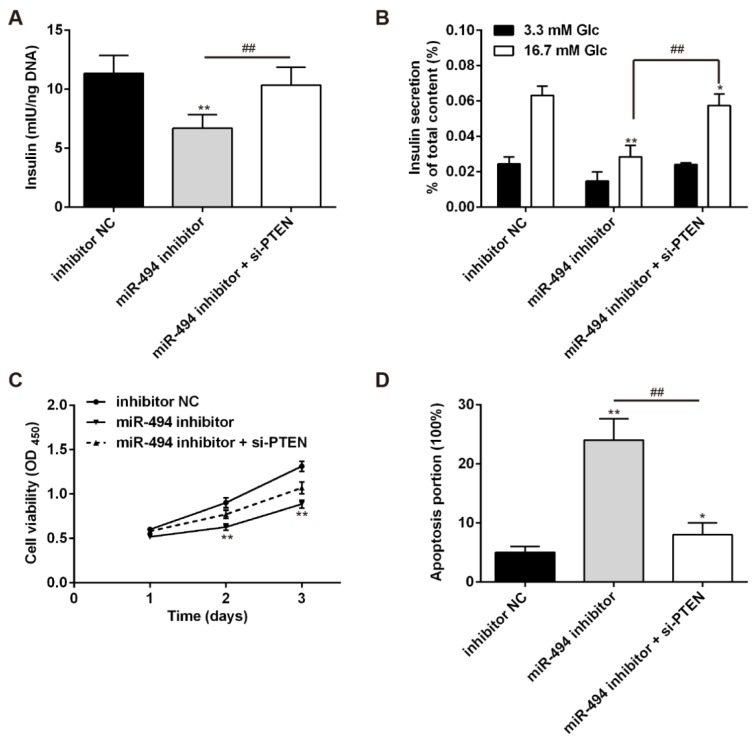
PTEN is involved in the inhibitory effects of miR-494 knockdown on functions of pancreatic β-cell. (A) INS-1 cells were co-transfected with miR-494 inhibitor and si-PTEN. 48 h after transfection, total insulin content was measured by ELISA assay. (B) Glucose-stimulated insulin secretion was then determined in each group. (C) MTT assay and (D) flow cytometry were then performed to examine cell proliferation and apoptosis. All data are expressed as the mean ± SD. **P < 0.01 vs inhibitor NC, ^##^P < 0.01 vs miR-494 inhibitor

To further investigate the relevance of miR-494/PTEN signaling in pancreatic β-cells dysfunction, we knocked down PTEN expression in INS-1 cells by siRNA. Then, we examined the glucose-stimulated insulin secretion, cell proliferation and cell apoptosis in INS-1 cells co-transfected with miR-494 inhibitor and si-PTEN. As shown in Figure 5A and B(Fig. 5), knockdown of PTEN restored the reduction of insulin secretion induced by miR-494 inhibition. Meanwhile, knockdown of PTEN increased cell viability and decreased the proportion of apoptosis mediated by miR-494 knockdown (Figure 5C and D(Fig. 5)). Taken together, these data demonstrate that downregulated miR-494 inhibits the proliferation and insulin secretion, while it induces the apoptosis of pancreatic β-cells by directly targeting PTEN.

## Discussion

In the present study, we found that miR-494 was down-regulated in peripheral blood from GDM patients. Furthermore, knockdown of miR-494 inhibited cell proliferation, insulin secretion and content, while induced apoptosis of pancreatic β function at least partly by targeting PTEN. These data suggest that miR-494/PTEN axis may be a potential therapeutic target for GDM.

Increasing evidence indicates that miRNAs are involved in diverse types of diabetes and a number of miRNAs have been reported to be involved in the regulation of pancreatic development and function (Latreille et al., 2015[17], Martinez-Sanchez et al., 2015[24]). For example, Li et al. (2016[19]) found that miR-19a-3p enhanced the proliferation and insulin secretion, while it inhibited the apoptosis of pancreatic β-cells via the inhibition of suppressor of cytokine signaling 3 (SOCS3). A study performed by Bao et al. showed that overexpression of miR-185 enhanced insulin secretion of pancreatic β-cells, promoted cell proliferation and protected cells from apoptosis (Bao et al., 2015[2]). Although many miRNAs have already been identified, their roles in the regulation of key genes and signaling pathways associated with GDM pathology still remain largely unknown. In this study, we retrieved a miRNA dataset (GSE19649) that displayed a set of differentially expressed miRNAs, including miR-494, which was down-regulated in blood and placenta from GDM patients. Moreover, a significant inverse correlation was observed between miR-494 level and the blood glucose concentration among the GDM patients. Our data imply its potential role in GDM. 

Previous studies have mainly focused on the role of miR-494 in human cancers. Zhao et al. demonstrated that miR-494 inhibited invasion and proliferation of gastric cancer by targeting IGF-1R (Zhao et al., 2016[40]). Moreover, miR-494 was reported to suppress cell proliferation and induced senescence in A549 lung cancer cells, suggesting a tumor suppressor role (Ohdaira et al., 2012[25]). By contrast, overexpression of miR-494 increased proliferation through an acceleration of G1/S transition by targeting the Mutated Colorectal Cancer (MCC) tumor suppressor in human hepatocellular carcinoma (Lim et al., 2014[20]), suggesting that it may play an oncogenic role in hepatocellular carcinoma. Our results, consistent with these conclusion derived from study based on cancer, showed that low-level of miR-494 inhibited the proliferation and insulin secretion, while it promoted the apoptosis of pancreatic β-cells, suggesting that different molecular network functioning in GDM compared with cancer.

Recently, many new insights into the core signaling pathways in GDM have been made, including AKT pathways (Hao et al., 2015[14]). Sun et al. found that overexpression of miR-494 increased the expression of HIF-1α through activating the PI3K/AKT signaling pathway and protected against hypoxia-induced apoptosis in the immortalized hepatocyte cell line L02 (Sun et al., 2013[32]). Moreover, a recent study showed that exendin-4 promoted β-cell proliferation via PI3k/AKT signaling pathway (Wang et al., 2015[36]). Therefore, the PI3K/AKT pathway may also be involved in the protective effects of miR-494 against pancreatic β-cell apoptosis. In this study, PTEN, a negative regulator of PI3K/ AKT pathway, was identified as a direct target of miR-494 in INS-1 cells. Furthermore, we disclosed that knockdown of miR-494 inhibited cell proliferation and insulin secretion, while it promoted the apoptosis of pancreatic β-cells by targeting PTEN through inactivation of PI3K/AKT pathway.

In conclusion, our results provide new insights that low-level miR-494 was involved in the progression of GDM through inhibiting pancreatic β-cells functions. Moreover, our data disclose a novel miR-494/PTEN signaling cascade in GDM, suggesting a possible candidate for GDM treatment.

## Acknowledgements

None.

## Conflict of interest

We all declare that we have no conflict of interest.
